# Studies on Indomethacin Intraocular Implants Using Different *in vitro* Release Methods

**DOI:** 10.4103/0250-474X.41458

**Published:** 2008

**Authors:** J. Balasubramaniam, A. Srinatha, J. K. Pandit

**Affiliations:** ISP (Hong Kong) Limited, H. No 6-3-1090/A, Bhupal Towers, Rajbhavan Road, Somajiguda, Hyderabad-500 082, India; 1 Department of Pharmaceutics, Institute of Technology, Banaras Hindu University, Varanasi-221 005, India

**Keywords:** Intraocular implants, dissolution, sodium alginate, indomethacin, *in vitro* release

## Abstract

Intra ocular implants of sodium alginate alone and in combination with hydroxypropylmethylcellulose with or without calcium chloride were formulated with indomethacin as a model drug. The drug release from the implants was evaluated using static method, continuous flow through apparatus (developed in house), USP dissolution and agar diffusion. Except in the static method, indomethacin particle size did not impart any effect on the drug release. In agar diffusion method, an increase in agar concentration from 1 to 2% resulted in a significant decrease (P< 0.005) in the amount of drug released. Inclusion of hydroxypropylmethylcellulose (33.3, 41.6 and 50% w/w), resulted in decrease of indomethacin release irrespective of the method of dissolution study. The agar diffusion method and the continuous flow through methods seem to simulate to a certain extent the *in vivo* conditions as far as the placement of the device and the hydrodynamic diffusion layer around the intra ocular implant is concerned. The static method and USP method affected the hydrodynamic diffusion layer either too slowly or too fast.

In ocular drug delivery systems, the primary but important efforts concern the conception and design of new biodegradable implantable systems to interior parts of the eye to prolong the residence time. Novel systems are designed to effectively target the ocular region like liposomes or microspheres, which are injected into the vitreous cavity with a fine needle^1^–^3^. However, intraocular devices have superior patient compliance than conventional systems of ocular administration of drugs.

The biodegradable polymeric implants aid in maintaining drug concentration in a therapeutically appropriate range thereby reducing harmful side effects associated with intra-vitreal and IV administration. These devices provide a continuous, long-term administration of drug thus eliminating the discomfort associated with multiple dosing and improve patient compliance^4^. Fabrication and evaluation of Intra ocular implants is still very tentative due to the highly complex nature of the tissue in which they are placed. The limited knowledge about the flow of ocular fluids around the implanted device requires that its *in vitro* release should be studied and optimized carefully.

*In vitro* dissolution plays an important role in the development process of a new formulation as it gives a preliminary data on the drug release behavior of the formulation. From the formulation viewpoint it is extremely important that at various stages of development, the formulator is able to test the release profile of the drug in an environment, which would closely mimic the *in vivo* condition. Agar diffusion, USP basket dissolution method, static method and continuous flow through apparatus are widely employed for * in vitro* drug release from intra ocular implants. There is seldom any investigation reporting a comparative evaluation and factors influencing these methods.

## MATERIALS AND METHODS

Indomethacin was generously gifted by Jagsonpal Pharmaceuticals, New Delhi (India), Sodium Alginate and hydroxypropylmethylcellulose (HPMC) K-100 was procured from Loba Chemie Pvt. Ltd., Mumbai, (India) and Dow Chemicals (USA), respectively. Agar was purchased from Hi-media Pvt. Ltd., Mumbai, (India). All other chemicals were of analytical grade.

### Fabrication of implants:

Indomethacin and sodium alginate alone and in combinations with HPMC, with or without calcium chloride were mixed together well in a mortar and slugged on a Manesty E_2_ (Manesty, U.K) single punch machine using 12 mm diameter punches. The resultant slugs were broken and sieved through sieve #60 and #100. The granules passing through #60 and retained over #100 were compressed directly using 2×7.5mm punches on Manesty E_2_ single punch machine to yield track-field type implants.

### Evaluation of implants:

For uniformity of weight, ten implants from each batch were weighed individually and their average determined. Friability and hardness were tested using Roche's friabilator (n=6) and Monsanto hardness tester (n=10). For determination of uniformity of drug content, 6 implants from each batch were weighed individually and dissolved in 50 ml of phosphate buffer (pH 7.4). The resultant solution was filtered through G2 glass filter. An aliquot of the filtrate was diluted suitably and analyzed for indomethacin content at 319.5 nm (Shimadzu, UV-1601, Japan).

### Dissolution studies by agar diffusion method:

Ten millilitres of 1 and 2% w/v sterilized agar solution was poured on Petri dishes (90 mm diameter), aseptically and allowed to set. A circular hole was made in the center of the agar plates and the implant was placed at the center of the bore and covered with the agar plug. The implants were removed at pre-determined time intervals and dissolved in phosphate buffer (pH 7.4), filtered, diluted and residual drug content was measured spectrophotometrically at 319.5 nm. The agar gel was dissolved in hot phosphate buffer (pH 7.4) and analyzed for indomethacin content using an appropriate blank.

### Dissolution studies using USP apparatus:

USP apparatus-I (Campbell Electronics, Mumbai) was used with 500 ml of phosphate buffer (pH 7.4) at 37±1° as the dissolution medium with the basket speed maintained at 50 rpm. At pre-determined time intervals aliquots were withdrawn and replaced with equal volume of pre-warmed buffer. The samples were analyzed for indomethacin content at 319.5 nm, spectrophotometrically.

### Dissolution studies by static method:

Individually weighed implants were placed in stainless steel mesh holder of dimensions 2×4×6 mm and suspended in amber coloured vials containing 10 ml of phosphate buffer (pH 7.4) and placed in a water bath thermostated at 37±1°. At pre-determined time intervals the dissolution medium was completely withdrawn and replaced with pre-warmed buffer to ensure sink conditions. The withdrawn samples were analyzed for indomethacin content as described earlier.

### Dissolution studies on flow-through apparatus:

The studies were conducted using an in house fabricated dissolution cell, described elsewhere^5^.

The dissolution cell consisted of two circular plates of 3.8 cm diameter and 1.2 cm thickness, made of acrylate. The plates were held together by means of 3 screws. The bottom plate had a groove of 1.7 cm diameter and 6 mm deep, fitted with #80 mesh for supporting the implant. An outlet tube was provided for collecting the eluate. The top plate had a hole for the inlet of the dissolution medium (phosphate buffer pH 7.4). The entire setup was connected from the top by a silicone tubing of 1mm internal diameter to a peristaltic pump. The flow rate of the medium was maintained at 0.8 ml/h and the eluate was collected in amber coloured vials as a function of time and analyzed for indomethacin content at 319.5 nm.

### Statistical evaluation:

Experimental results are expressed as mean ± standard deviation (SD). The student ‘t’ test was performed to determine the level of significance. Differences were considered to be statistically significant at *P*<0.05

## RESULTS AND DISCUSSION

The formulation variables and the physico-chemical characteristics of the various batches of the prepared implants are shown in Tables 1 and 2, respectively. Thickness, weight and drug content varied within ± 5%. Kunou *et al*^6^,^7^, studied the in vitro release from PLGA scleral implants of gancyclovir by incubating the implants in 2 ml of phosphate buffered solution in a shaking water bath at 37°. Balasubramaniam *et al*^4^,^8^, reported a static method for studying the *in vitro* release of indomethacin from film type intra ocular implants of indomethacin. As there is a lot of variation in the different methods used by various investigators, an attempt was made in the present study to evaluate the dissolution profiles of the prepared (compressed) implants using four different methods. In all the cases effect of parameters like particle size of the drug, HPMC concentration and calcium chloride concentration were studied.

**TABLE 1:** COMPOSITION OF SODIUM ALGINATE IMPLANTS (FORMULA/ IMPLANT)

Batch code	Sodium alginate (mg)	Calcium chloride (mg)	HPMC K-100 (mg)	Indomethacin (mg)
BS1	25	-	-	5
BS2	25	-	-	5
BS3	12.5	-	12.5	5
BS4	10	-	15	5
BS5	20	5	-	5
BS6	15	-	10	5
BS7	17.5	7.5	-	5
BS8	15	10	-	5

**TABLE 2:** PHYSICO-CHEMICAL PROPERTIES OF THE PREPARED IMPLANTS

Batch code	Weight (mg±SD)	Thickness (mm±SD)	Hardness (Kg)	Friability (%±SD)	DCU* (%±SD)
BS1	29.2±0.48	0.54±0.001	7.5±0.48	0.90±0.01	97.5±0.56
BS2	28.5±0.67	0.52±0.08	7.5±0.34	0.94±0.04	98.4±0.23
BS3	30.1±0.44	0.55±0.004	8.0±0.18	0.92±0.02	99.8±0.35
BS4	29.7±0.18	0.54±0.006	7.5±0.66	0.92±0.05	98.7±0.58
BS5	30.5±0.18	0.58±0.004	8.0±0.48	0.92±0.03	100.01±0.14
BS6	29.8±0.44	0.55±0.006	7.0±0.81	0.90±0.04	97.9±0.14
BS7	28.9±0.81	0.53±0.004	7.5±0.11	0.94±0.03	99.4±0.21
BS8	29.7±0.74	0.54±0.002	7.5±0.41	0.90±0.02	99.6±0.48

* DCU- Drug content uniformity

Drug release from all the methods followed square root of time kinetics, as determined by correlation coefficient values. Further, the release exponent (n) values were also predominantly suggestive of matrix diffusion kinetics, excepting the agar diffusion method, wherein the ‘n’ values suggested the prevalence of an anomalous (erosion) mechanism in addition to the swelling controlled (matrix) diffusion.

**Fig. 1:**
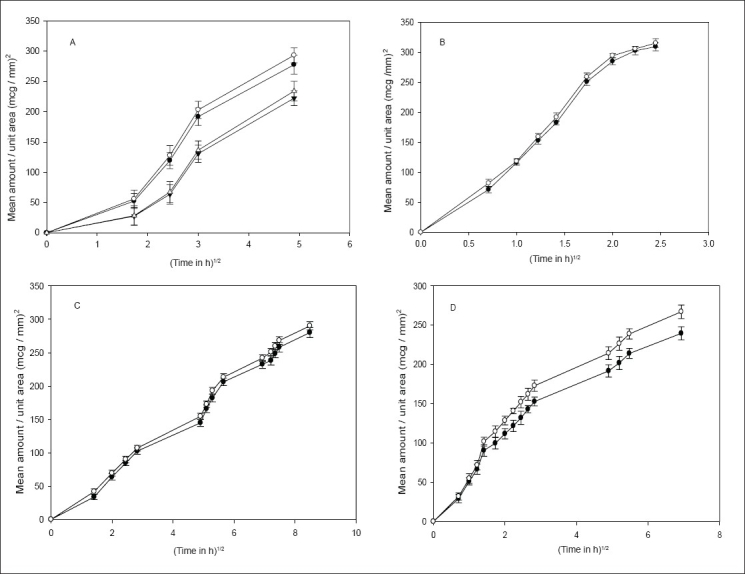
Effect of particle size on indomethacin release. A – Agar diffusion (1%) [(–●–) BS2; (–◦–) BS3; (–▼–) BS4; (–Δ–) BS6]; B – USP method [(–●–) BS2; (–◦–) BS3; (–▼–) BS4; (–Δ–) BS6]; C – continuous flow–through apparatus [(–●–) BS2; (–◦–) BS3; (–▼–) BS4; (–Δ–) BS6] and D – static method [(–●–) BS2; (–◦–) BS3; (–▼–) BS4; (–Δ–) BS6].

**Fig. 2:**
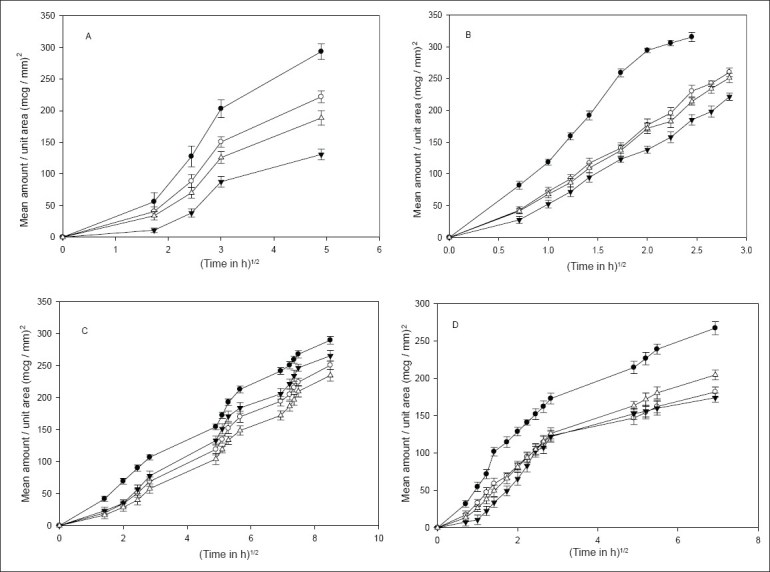
Influence of HPMC concentration on drug release. A – Agar diffusion (1%) [(–●–) BS2; (–◦–) BS3; (–▼–) BS4; (–Δ–) BS6] B – USP method [(–●–) BS2; (–◦–) BS3; (–▼–) BS4; (–Δ–) BS6]; C – continuous flow–through apparatus [(–●–) BS2; (–◦–) BS3; (–▼–) BS4; (–Δ–) BS6] and D – static method [(–●–) BS2; (–◦–) BS3; (–▼–) BS4; (–Δ–) BS6].

**Fig. 3:**
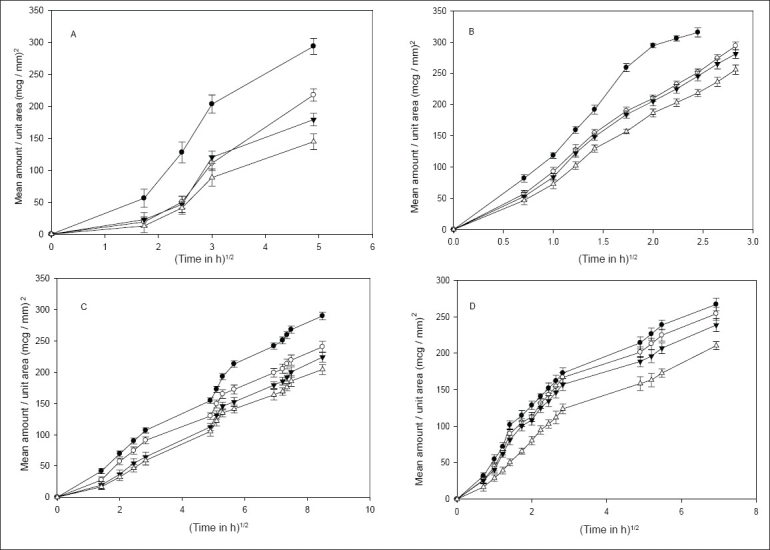
Effect of implant cross–linking with calcium chloride on drug release. A – Agar diffusion (1% Agar) [(–●–) BS2; (–◦–) BS5; (–▼–) BS7; (–Δ–) BS8]; B – USP method [(–●–) BS2; (–◦–) BS5; (–▼–) BS7; (–Δ–) BS8]; C – continuous flow–through apparatus [(–●–) BS2; (–◦–) BS5; (–▼–) BS7; (–Δ–) BS8] and D – static method [(–●–) BS2; (–◦–) BS5; (–▼–) BS7; (–Δ–) BS8].

Drug release was independent of the particle size of the drug from all the methods except the static method, where a significant difference (*P*<0.05) was observed (fig. 1). Fig. 2 shows that, an increase in the concentration of HPMC in the implants caused a significant decrease in the release rate of indomethacin. Though various factors are likely to be responsible for the decrease in drug release with an increase in HPMC concentration from all the methods, some general trend seems to appear. HPMC partially hydrates forming a pseudo gel which control swelling of the implant, subsequently overall dissolution rate and drug availability. Once the protective gel layer is formed, two rate mechanisms predominate^9^. Firstly, the pseudo gel permits additional water to penetrate into the device, extending the gel layer into the implant. Secondly, the outer gel layer fully hydrates and begins to be dissolved by the fluids. For sparingly soluble drugs like indomethacin, the dissolution rate is primarily dependent on diffusion (due to swelling) and to some extent on erosion, which in turn is dependent on viscosity of the gel. Thus increasing the concentration of HPMC increases the viscosity of the resultant gel, thus resulting in slower drug release. Cross-linking of sodium alginate involves the interaction between cations and ‘G-residues’ in sodium alginate, resulting in the formation of an ‘egg box’ structure^10^. (fig. 3), indicate that an increment of calcium chloride caused a significant decrease (*P*<0.05) in the amount of drug released from all the methods. With increasing concentration of the cross-linker (Ca^ ++^ ), more calcium ions will be available for cross-linking, which results in the formation of a dense calcium alginate matrix that can bring about a decrease in drug release.

Sodium alginate, swells on contact with aqueous dissolution medium resulting in an increase in the porosity of the matrix, thus facilitating the mobilization of the water molecules into the polymer matrix. The dissolution medium dissolves the calcium chloride incorporated in the sodium alginate implants, resulting in an *in situ* cross-linking of sodium alginate, which is an instantaneous phenomenon. In agar diffusion and continuous flow through methods, the amount of aqueous medium that contacted the implant was significantly less and the time of contact between the implant and the dissolution medium was comparatively higher than the other two methods, thus the cross-linking of the sodium alginate matrix in these two methods would be progressive and more uniform in comparison to the static method, wherein the presence of relatively more dissolution medium would have resulted in rapid, but incomplete cross-linking. In USP dissolution method the implant disintegrated upon swelling which resulted in formation of isolated cross-linked mass.

An increase in the concentration of agar from 1 to 2% resulted in a significant decrease (*P*<0.05) in the amount of drug released which may be a consequence of increase in the strength of the gel formed. The increment in gel strength decreases the amount of available aqueous medium, as the water molecules would be entrapped within the gel structure, resulting in decreased rate of diffusion of the drug from the implants to the surrounding medium. It has been reported earlier that the drug diffusion coefficients from HPMC and sodium alginate matrices are strongly dependent on the water content of the system^11^.

Amongst the methods studied, the agar diffusion method and the continuous flow through method seem to have the potential to prolong the drug release from the compressed implant and also simulate to certain extent the *in vivo* conditions as far as the placement of the device is concerned. The static method, which was successfully utilized for evaluating drug release kinetics from film type implants^4^,^8^ was not suitable for evaluating drug release from the compressed implant, since the mesh holder, in which the implant was placed acted as an impediment to the uniform swelling of the implant, resulting in irregular swelling during the course of the study.
